# Thyroid Parenchyma Microcalcifications on Ultrasound for Predicting Lymph Node Metastasis in Papillary Thyroid Carcinoma: A Prospective Multicenter Study in China

**DOI:** 10.3389/fonc.2021.609075

**Published:** 2021-03-03

**Authors:** Juan Liu, XiaoHong Jia, Ying Gu, Xia Chen, Ling Guan, JiPing Yan, Hong Zhai, Na Zhou, YiJie Dong, WeiWei Zhan, XiaoMao Luo, JianQiao Zhou

**Affiliations:** ^1^Department of Ultrasound, Ruijin Hospital, Shanghai Jiaotong University School of Medicine, Shanghai, China; ^2^Department of Ultrasound, Affiliated Hospital of Guizhou Medical University, Guiyang, China; ^3^Department of Ultrasound, Gansu Provincial Cancer Hospital, Lanzhou, China; ^4^Department of Ultrasound, Shanxi Provincial People's Hospital, Taiyuan, China; ^5^Department of Ultrasound, Affiliated Hospital of T.C.M of Xinjiang Medical University, Urumqi, China; ^6^Department of Ultrasound, Yunnan Cancer Hospital, Kunming, China

**Keywords:** papillary thyroid carcinoma, microcalcifications, lymph node metastasis, psammoma bodies, ultrasound

## Abstract

**Objective:** Cervical lymph node metastasis (LNM) was found to be clinically significant prognostic factors of patients with papillary thyroid carcinomas (PTC). Ultrasound (US) characteristics of thyroid nodules and thyroid parenchyma may be used to predict LNM. To investigate the value of nodular US features as well as thyroid parenchymal microcalcifications on US in predicting LNM in patients with PTC.

**Methods:** This prospective study was approved by the Institutional Review Board. From January 2018 to June 2019, 971 consecutive patients with solitary PTC who underwent preoperative neck US evaluation were included from six hospitals in China. The US features of thyroid nodules as well as thyroid parenchyma microcalcifications were carefully evaluated based on the static images and dynamic clips. Univariate and multivariate analyses were performed to determine independent predictors of LNM.

**Results:** Of the 971 patients, 760 were female, 211 were male. According to the pathological examination, 241(24.82%) patients were found with cervical LNM (LNM positive group), while 730 (75.18%) patients were not (LNM negative group). Multiple logistic regression analysis showed that young age (<55 years old) (OR = 1.522, *P* = 0.047), large size (>10 mm) (OR = 1.814, *P* < 0.001), intratumoral microcalcifications (OR = 1.782, *P* = 0.002) and thyroid parenchyma microcalcifications (OR = 1.635, *P* = 0.046) were independent risk factors for LNM of PTC.

**Conclusions:** Young age, large nodule size, intratumoral microcalcifications, as well as thyroid parenchyma microcalcifications on US are independent predictors of cervical LNM for patients with PTC.

## Introduction

Although it accounts for <3% of all cancers, thyroid cancer is the most common endocrine malignancy (1, 2). In recent years, its incidence has continuously increased all over the world (3–5). Differentiated thyroid cancer comprises the vast majority of all thyroid cancers and the most common type is the papillary thyroid carcinoma (PTC) which accounts for ~80–90% of all primary thyroid cancers (5–7). Regardless of the fact that PTC has a good prognosis and distant metastases are rare, its high incidence of central or lateral cervical lymph node metastasis (LNM) is a cause for concern (8). PTC can occasionally lead to death due to recurrence and metastasis. LNM was the main risk factor for local recurrence and distant metastasis of thyroid carcinoma (9–12), and was found to be clinically significant prognostic factors of patients with PTC (13, 14).

Preoperative ultrasonography (US) has been recognized as the most useful tool for diagnosing LNM of PTC (15). Accurate preoperative US evaluation helps to develop strategies for complete resection of the primary PTC and the involved cervical lymph nodes. However, there is controversy regarding the value of preoperative US for the diagnosis of LNM. Some studies have obtained optimistic results, showing that the sensitivity and specificity of preoperative US in detecting central LNM were 62–95% and 75–90%, respectively (16, 17). However, more studies have shown that the sensitivity of US in detecting central LNM was unsatisfactory (18–22). To improve the diagnostic efficacy of US for central LNM, previous studies have investigated clinical factors or thyroid nodule US features in patients with PTC to predict LNM. Features such as age, sex, tumor size, the distance between the tumor and the capsule, and microcalcifications play an important role in predicting lymph node malignancy (23–27). However, most of previous studies have ignored the potential predictive value of ultrasonic features of thyroid parenchyma.

As early as 1980s, a pathological study carried out by Johannessen et al. (28) revealed that thyroid psammoma bodies (PBs) could be found in the thyroid parenchyma adjacent to the thyroid papillary carcinomas. These PBs represent calcified metastatic tumor thrombi in the thyroid lymphatics. Moreover, similar intralymphatic PBs were also found in some regional metastatic lymph nodes. It has been pointed out in the literature that PBs in the thyroid parenchyma are common in papillary thyroid carcinomas with a high degree of invasion (29). Therefore, it is reasonable to assume that it is possible to predict the LNM of PTC through the imaging of PBs in the thyroid parenchyma. On US, PBs appear as tiny punctate hyperechoic foci without posterior acoustic shadowing, indicative of microcalcifications (30, 31).

The purpose of the present study was to investigate the value of thyroid parenchymal microcalcifications on US in predicting LNM, based on a prospective multicenter study with 971 PTC patients.

## Materials and Methods

### Design and Overview

This multicenter, prospective study was performed from January 2018 to June 2019. This study was approved by the Ethics Committee of Ruijin Hospital, Shanghai Jiao Tong University School of Medicine and written informed consent was obtained from all participants for this prospective study. Patients who underwent lobectomy or total thyroidectomy plus central lymph node dissection for solitary PTC were collected from six hospitals in China, of which lateral lymph node dissection was selectively performed in 97 patients with lateral lymph node metastasis diagnosed by fine needle aspiration (FNA).

Inclusion criteria: (1) pre-surgery diagnosis of PTC achieved by combined US and US-guided FNA; (2) central lymph node dissection was performed; (3) for patients with suspicious lateral lymph nodes detected by US and/or CT and subsequently confirmed as malignant by fine needle aspiration biopsy, central group lymph node plus lateral lymph node dissection was performed; (4) cases with complete clinical data (including US images, pathological results etc.).

Exclusion criteria: cases with excessive tumor volume that could not be evaluated for characteristics of thyroid parenchyma were excluded.

### Image Acquisition

All of the patients were subjected to preoperative US examinations with a variety of commercially available high-end US systems equipped with high-frequency transducers. In each sub center, the preoperative US examination was performed by radiologists with more than 5 years of experience in thyroid US. Before the start of the study, all radiologists were uniformly trained to standardize the thyroid and cervical lymph node scan methods and image acquisitions according to the AIUM practice guideline for performing thyroid US (32). In particular, US clip recording the dynamic US information from the upper pole to the lower pole of the ipsilateral thyroid gland was acquired for each patient. All images and clips were stored on the local hard disk first, and then copied to the mobile hard disk and sent to the central hospital for unified analysis.

### Image Analysis

The features of the target nodule and the thyroid parenchyma were assessed by two blinded professionally trained radiologists (J.L. and X.H.J) with 6 and 10 years of experience in thyroid US in consensus. In cases of discordance in the evaluation between the two radiologists, a third radiologist (J.Q.Z) with 20 years of experience in thyroid US make the final decision.

The US features of thyroid nodules assessed included size (the maximum diameter: >10 mm or ≤ 10 mm), orientation (taller-than-wide or wider-than-tall), margin (circumscribed, irregular, and ill-defined), halo (present or absent), composition (solid, predominately solid, and predominately cystic), echogenicity (hypoechoic, isoechoic, hyperechoic, and markedly hypoechoic), echotexture (homogeneous or heterogeneous), echogenic foci (microcalcifications, macrocalcifications, peripheral calcifications, and no echogenic foci), posterior features (enhancement, shadowing, combined pattern, and no posterior features), and contact extent between the nodule border and thyroid capsule(0–50, 50–100%).

In particular, thyroid parenchyma microcalcifications, defined as punctate hyperechoic foci without posterior acoustic shadowing in the extratumoral area of the thyroid parenchyma, were carefully evaluated based on the static images and dynamic clips. In additional, the echotexture of the thyroid parenchyma was divided into homogeneous and heterogeneous (33).

### Statistical Analysis

The chi-square test was first used to compare US findings between LNM positive and LNM negative groups. For evaluating the relationship between US features and LNM status, the second step was to build a multiple logistic model with forward stepwise selection to determine independent predictors of LNM. Significant variables were included in the final logistic regression analysis. The selection criteria alpha was set to 0.05. Odds ratios (ORs) and 95% confidence intervals (CIs) for each US feature were obtained from the logistic regression model. The analysis was performed using the SAS software (version 9.4; SAS Institute, NC, USA). A *P* < 0.05 was considered as indicating statistical significance.

## Results

### Clinical Characteristics of Patients

A total of 971 patients with 971 PTC were eventually included (Table 1). Of the 971 patients, 760 were female patients, 211 were male patients. Patients ranged in age from 11 to 76 years (mean, 43.9 ± 11.4 years). The age of male patients (41.4 ± 11.0, 11–73 years) was significantly lower than that of the female patients (44.6 ± 11.4, 16–76 years; *P* < 0.001). The mean PTC nodule size was 12.46 ± 8.53 mm (range, 2.7–71.8 mm). According to the pathological examination after thyroid carcinoma surgery, 241 (24.82%) patients were found with cervical LNM (LNM positive group), while 730 (75.18%) patients were not (LNM negative group). The mean nodule size of the LNM positive group was 14.98 ± 9.32 mm (range, 3.0–53.0 mm), which was significantly larger than that of the LNM negative group (11.63 ± 8.09 mm, 2.7–71.8 mm; *P* < 0.001).

**Table 1:** Clinical characteristics of patients.

**Category**	**Total (*n* = 971)**
**Gender**	
Male	211 (21.73%)
Female	760 (78.27%)
**Age, years (mean ± SD)**	
Total	43.9 ± 11.4
Male	41.4 ± 11.0
Female	44.6 ± 11.4
**Lymph nodes metastasis**	
Absent	730 (75.18%)
Present	241 (24.82%)
**Tumor size, mm (mean ± SD)**	
Total	12.46 ± 8.53
LNM positive group	14.98 ± 9.32
LNM negative group	11.63 ± 8.09

### Univariate Analysis of Factors in PTC With LNM

Patient demographics and US features of the thyroid nodules and thyroid parenchyma in the LNM positive and negative groups were presented in Table 2. The composition, halo and posterior features of thyroid nodules did not reach statistical significance between the LNM positive and negative groups (*P* > 0.05 for all), as well as the echotexture of the thyroid parenchyma (*P* = 0.674). In contrast, gender, age, nodule size, orientation, margin, echogenicity, echotexture, echogenic foci, contact with the adjacent capsule, and thyroid parenchyma microcalcifications were statistically significantly different between the two groups (*P* < 0.05 for all). Male, young age (<55 years old), large nodule size (>10 mm), wider-than-tall orientation, ill-defined or irregular margin, hypoechoic, heterogeneous echotexture, intratumoral microcalcifications, more than 50% contact with the adjacent capsule, and thyroid parenchyma microcalcifications were more common in the LNM positive group than in the LNM negative group (*P* < 0.05 for all).

**Table 2:** Patient demographics and ultrasound features in the LNM positive and negative groups.

**Category**	**Univariate study**		
	**LNM positive group *N*(%)** **(*n* = 241)**	**LNM negative group *N*(%)** **(*n* = 730)**	***P-*value**
**Gender**			
Male	69(32.70%)	142(67.30%)	0.003
Female	172(22.63%)	588(77.36%)	
**Age (years)**			
<55	207(26.37%)	578(73.63%)	0.022
≥55	34(18.28%)	152(81.72%)	
**Orientation**			
Wider-than-tall	171(27.36%)	454(72.64%)	0.014
Taller-than-wide	70(20.23%)	276(79.77%)	
**Size(mm)**			
≤ 10	88(18.00%)	401(82.00%)	<0.001
>10	153(31.74%)	329(68.26%)	
**Margin**			
Circumscribed	3(7.14%)	39(92.86%)	0.019
Irregular	199(25.16%)	592(74.84%)	
Ill-defined	39(28.26%)	99(71.74%)	
**Halo**			
Absent	231(25.41%)	678(74.59%)	0.102
Present	10(16.13%)	52(83.87%)	
**Composition**			
Solid	226(24.38%)	701(75.62%)	0.145
Predominately solid or Predominately cystic	15(34.09%)	29(65.91%)	
**Echogenicity**			
Markedly hypoechoic	18(10.06%)	161(89.94%)	<0.001
Hypoechoic	213(28.36%)	538(71.64%)	
Isoechoic or Hyperechoic	10(24.39%)	31(75.61%)	
**Echotexture**			
Homogeneous	71(20.58%)	274(79.42%)	0.023
Heterogeneous	170(27.16%)	456(72.84%)	
**Echogenic foci**			
Microcalcifications	162(31.15%)	358(68.85%)	<0.001
Macrocalcifications or peripheral calcifications	25(19.38%)	104(80.62%)	
No echogenic foci	54(16.77%)	268(83.23%)	
**Posterior features**			
Enhancement	19(27.14%)	51(72.86%)	0.897
Shadowing	57(23.65%)	184(76.35%)	
No posterior features	162(25.12%)	483(74.88%)	
Combined pattern	3(20.00%)	12(80.00%)	
**Contact with the adjacent capsule**			
0–50%	201(23.73%)	646(76.27%)	0.040
50–100%	40(32.26%)	84(67.74%)	
**Thyroid parenchyma echotexture**			
Homogeneous	208(25.06%)	622(74.94%)	0.674
Heterogeneous	33(23.40%)	108(76.60%)	
**Thyroid parenchyma microcalcifications**			
Absent	207(23.31%)	681(76.69%)	<0.001
Present	34(40.96%)	49(59.04%)	

### Multivariate Analysis of Factors in PTC With LNM

**Figure 1:**
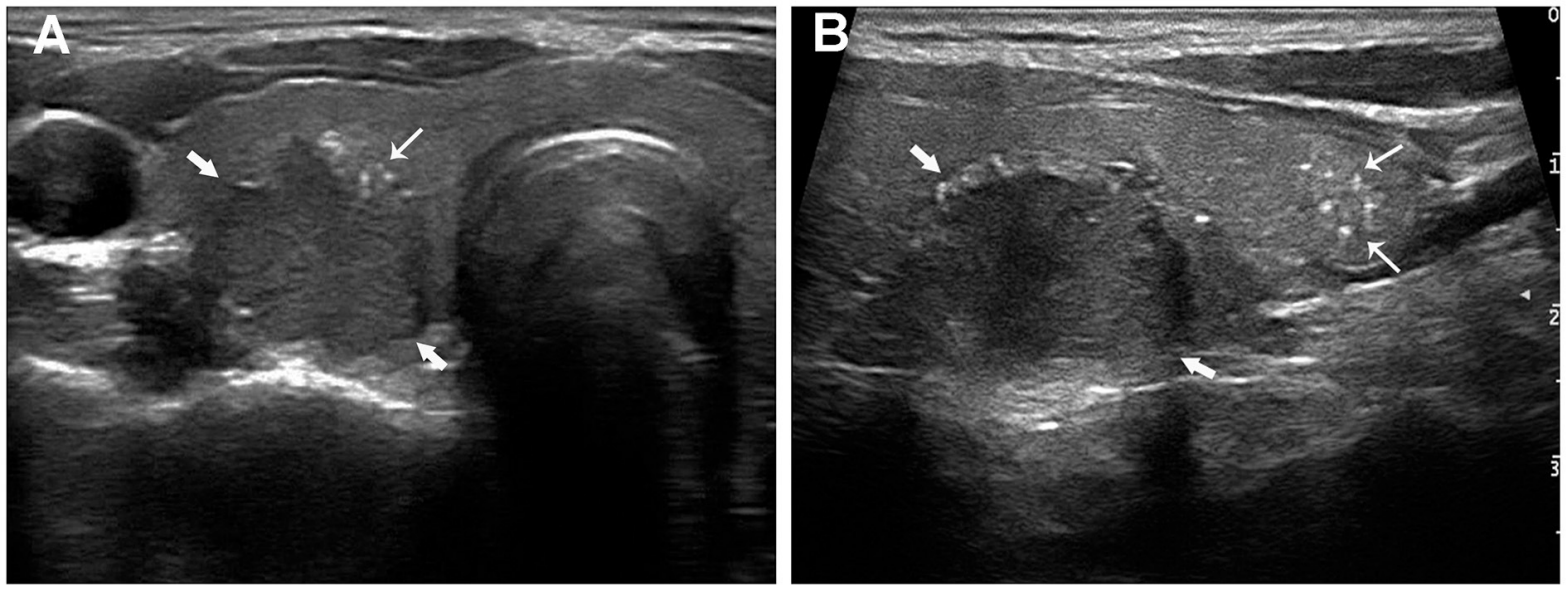
Papillary thyroid carcinoma in a 24-year-old woman with LNM. The transverse section **(A)** showed a 20.4 mm hypoechoic nodule (thick arrows) with intratumoral microcalcifications (fine arrows). The longitudinal section **(B)** showed multiple thyroid parenchyma microcalcifications (fine arrows) inferior to the nodule (thick arrows).

**Figure 2:**
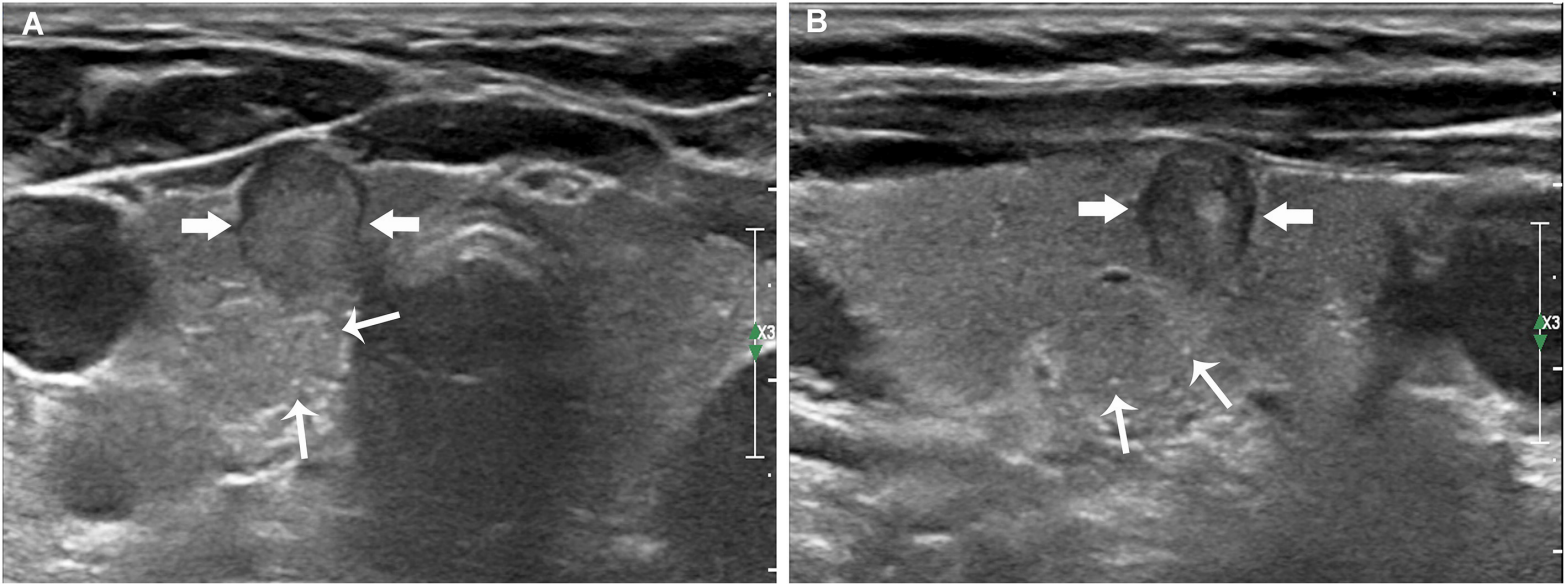
Papillary thyroid carcinoma in a 59-year-old woman without LNM. The transverse **(A)** and longitudinal **(B)** sections showed an 8.6 mm hypoechoic nodule (thick arrows) without intratumoral microcalcifications. Multiple thyroid parenchyma microcalcifications (fine arrows) appeared on the outside of the nodule.

The multiple logistic regression analysis showed four features were significantly associated with cervical LNM (Table 3). Young age (<55 years old) (OR = 1.522), large nodule size (>10 mm) (OR = 1.814), intratumoral microcalcifications (OR = 1.782), and thyroid parenchyma microcalcifications (OR = 1.635) could independently predictive of the presence of LNM (Figures 1, 2).

**Table 3:** Odds ratios for patient demographics and the US features by multivariate logistic regression.

**Category**	**Logistic regression**	
	**Odds ratio (95% CI^**†**^)**	***P*-value**
**Age (years)**		
<55	1.522(1.005–2.304)	0.047
≥55	1.00 (reference)	
**Size(mm)**		
≤ 10	1.00 (reference)	
>10	1.814 (1.325–2.485)	<0.001
**Echogenic foci**		
Microcalcifications	1.782(1.239–2.562)	0.002
Macrocalcifications or peripheral calcifications	1.079(0.63–1.847)	0.781
No echogenic foci	1.00 (reference)	
**Thyroid parenchyma microcalcifications**		
Present	1.635 (1.008–2.65)	0.046
Absent	1.00 (reference)	

† *CI, Confidence interval*.

## Discussion

Although some reports suggested that cervical lymph node metastasis has no impact on the prognosis of low-risk PTC, studies based on large sample showed that cervical lymph node metastases were independent risk in patients with PTC (14, 34). Especially for older patients with PTC, elective central neck dissection may improve locoregional control and may reduce morbidity in the long term (35). Consequently, for patients with cN1 PTC, therapeutic lymph node dissection has been considered the standard treatment (36). US has been regarded as the most useful tool for diagnosing cervical LNM, and adequate preoperative US detection of regional lymph node is likely to change the surgical planning for patients with thyroid cancer (15, 36, 37). US has high sensitivity and specificity for the diagnosis of lateral LNM, thus providing reliable information to assist in surgical management (38). However, although the diagnostic specificity of US for central LNM was satisfactory, the sensitivity was only 10.5–38% (18–20, 22). Moreno et al. (21) found that in PTC, patients with a US negative central compartment LNM who underwent central compartment dissection, 71.4% of them were found to be histopathologically LNM. As a result, many guides have pointed out the inadequacy of US for the assessment of central compartment LMN in PTC (15, 36, 39). The main reasons influencing sensitivity include practitioner expertise, scanning skills, the obstruction of the trachea or esophagus, and the micro-metastases (<2 mm in diameter) of positive central neck nodes (36, 39). Moreover, metastatic lymph nodes in the central compartment lack the typical US characteristics of LNM such as microcalcifications, cystic appearance, focal hyperechoic area, and increased blood supply (36). Therefore, many patients with central LNM were categorized into clinically node-negative. Thus, in order to achieve personalized treatment, it is necessary to explore more US signs that can assist in cervical LNM prediction in patients with PTC.

Previous studies that predicted cervical LNM of PTC patients have focused on the nodule US characteristics (23–27). The association between US features classified by the thyroid imaging reporting and data system (TIRADS) and cervical LMN was also explored. In a study carried out by Park et al. (40), it was found that the number of suspicious TIRADS US features was associated with lateral LMN in PTC. However, to our knowledge, no previous studies have addressed the relationship between thyroid parenchymal US features and cervical LNM. In this study, both nodule US features and thyroid parenchyma US features were evaluated simultaneously preoperatively to predict cervical LNM in patients with PTC, which are important highlights of this study. Multivariate analysis demonstrated that in addition to young age (<55 years old), large tumor size (>10 mm), and intratumoral microcalcifications, thyroid parenchyma microcalcifications were also independent predictors of LNM in PTC. However, other thyroid nodule US features such as orientation, margin, echogenicity, echotexture, contact with the adjacent capsule, and thyroid parenchyma echotexture were not independently associated with LNM.

Features such as age and tumor size are known to be important clinical factors for patients with PTC. In agreement with previous studies (25, 41), we found that young patients (<55 years old) suffer a higher risk of LNM. Therefore, for young patients with PTC, the cervical lymph nodes need to be evaluated more carefully and thoroughly by US. Previous reports indicated that the primary tumor size was one of the independent risk factors for LNM (12, 24). In thyroid carcinoma, the primary tumor size determines the tumor stage which is associated with unlimited cell proliferation, as it increases, the possibility of LNM increases. We divided the tumor into two groups: >10 mm and ≤ 10 mm in this study. Results revealed that lesions with maximum diameter >10 mm (OR = 1.814) have a higher risk of LNM.

Microcalcifications were the highly specific signs and important US findings of suspicious malignant nodules (42, 43). Microcalcifications were defined as punctate bright echoes with or without acoustic shadowing which usually correspond to PBs and were suggestive of PTC and LNM (31, 44). Thyroid PBs in the tumor and non-tumorous areas may be formed by vascular thrombosis, calcification, and tumor cell necrosis or necrosis and calcification in intra-lymphatic tumor thrombi (28, 45). Our finding is consistent with previous reports (12, 27), nodules with microcalcifications were associated with LNM and thus, could be a useful predictive marker for LNM.

In this study, we attempted to explore the relationship between thyroid parenchyma microcalcifications and lymph node metastasis. The result showed a significant relationship between thyroid parenchyma microcalcifications and LNM. Hirokawa et al. (46) have found that the presence of PBs in normal thyroid tissue is related to LNM. PBs are probably associated with the spread of tumor cells via vascular or lymphatic channels in thyroid parenchyma, significantly correlated with LNM (29, 47). The presence of PBs may represent an active biologic process, and can be identified cytologically or ultrasonographically. Thus, we recommend that US identification of thyroid parenchyma microcalcifications may be a useful predictive indicator for LNM. That is, if thyroid parenchyma microcalcifications are found by US, the lymph node status should be assessed carefully. According to this study, by assessing the nodule US characteristic and the thyroid parenchyma microcalcifications, combined with an exhaustive US examination of the central compartment, the cN0 status of PTC patients can be determined more accurately, thus avoiding unnecessary central neck dissection to prevent the corresponding morbidity.

This study had several limitations. First, multifocal thyroid cancer was not included in this study. Second, the relationship between microcalcifications and PBs was not further confirmed by pathology. Third, although the evaluation of the ultrasound images was done at the center, the pathologic diagnosis of LNM was done independently at each sub-center, which may lead to differences in the pathologic findings. Fourth, the dissection of lateral neck lymph nodes was only based on preoperative imaging and FNA, which will miss patients with occult metastases in the lateral neck and thus lead to underestimation of the prevalence of lateral LNM.

In conclusion, young age (<55 years old), large size (>10 mm), intratumoral microcalcifications and thyroid parenchyma microcalcifications are independent predictors of cervical LNM. When these US features appeared, we should consider that the possibility of LNM may increase.

## Data Availability Statement

The raw data supporting the conclusions of this article will be made available by the authors, without undue reservation.

## Ethics Statement

The studies involving human participants were reviewed and approved by the Ethics Committee of Ruijin Hospital, Shanghai JiaoTong University School of Medicine. The patients/participants provided their written informed consent to participate in this study.

## Author Contributions

JQZ designed the research and revised the manuscript for important intellectual content. JQZ, JL, and XHJ participated in the analysis of ultrasonic images, analyzed the data, and wrote the paper. All the authors gave their final approval for the submission of this version and any revised version for publication.

## Conflict of Interest

The authors declare that the research was conducted in the absence of any commercial or financial relationships that could be construed as a potential conflict of interest.
